# The Efficacy of Washed Microbiota Transplantation on *Helicobacter pylori* Eradication: A Pilot Study

**DOI:** 10.1155/2020/8825189

**Published:** 2020-10-19

**Authors:** Zhi-Ning Ye, Harry Hua-Xiang Xia, Ran Zhang, Lan Li, Li-Hao Wu, Xu-Juan Liu, Wen-Rui Xie, Xing-Xiang He

**Affiliations:** ^1^Department of Gastroenterology, The First Affiliated Hospital of Guangdong Pharmaceutical University, Guangzhou 510080, Guangdong Province, China; ^2^Research Center for Engineering Techniques of Microbiota-Targeted Therapies of Guangdong Province, The First Affiliated Hospital of Guangdong Pharmaceutical University, Guangzhou 510080, Guangdong Province, China

## Abstract

**Aim:**

The fecal microbiota transplantation by washed preparation was recently coined as washed microbiota transplantation (WMT). This pilot study is aimed at exploring the feasibility and efficacy of WMT on *Helicobacter pylori* eradication.

**Methods:**

Consecutive patients who had been treated with WMT for various indications and who were positive for *H. pylori* infection before WMT treatment but had never received eradication therapy for *H. pylori* infection were invited to take a follow-up ^13^C-urea breath test. The associations of demographic, clinical factors, and laboratory indicators for gastric function and intestinal barrier function with the therapeutic effect were determined.

**Results:**

A total of 32 eligible patients were included, and the overall *H. pylori* eradication rate was 40.6% (13/32). Patients with *H. pylori* eradication had a higher pepsinogen ratio (PGR) than those without (13.00 ± 6.97*vs.*8.31 ± 3.733; *P* = 0.02). Female patients had a higher, albeit not statistically significant, eradication rate than male patients (53.85% *vs.* 31.58%; *P* = 0.208). Compared with lower gastrointestinal tract delivery route, middle gastrointestinal tract delivery route seems to be a more suitable way for the treatment of *H. pylori* infection (58.33% vs 16.67%; *P* = 0.152). There was no significant difference in other demographic and clinical factors between patients with and without *H. pylori* eradication.

**Conclusion:**

*H. pylori* infection is eradicated in a proportion of patients who have received WMT. An increased pre-WMT PGR appears to be associated with the therapeutic effect. Further studies are required to confirm the efficacy of WMT, especially in combination with currently recommended regimens in randomized controlled trials.

## 1. Introduction

*Helicobacter pylori* is a type of microaerophilic, spiral-shaped, Gram-negative bacteria, which is colonized in the human stomach and easily resists the extreme environment of gastric acid [1]. It is a major pathogen of chronic gastritis, peptic ulcer, and gastric cancer and is also associated with irritable bowel syndrome [2, 3]. Early in 1994, the World Health Organization defined *H. pylori* as a class I carcinogen of gastric cancer, which accounted for 90% of noncardiac cancer cases [4]. It is estimated that about 50% of the population worldwide is infected with *H. pylori*, and the prevalence in developing countries is much higher than that in developed countries [5]. It is well known that *H. pylori* infection is difficult to eradicate naturally without drug intervention [6]. Triple therapy consisting of a bismuth salt or proton pump inhibitors and two antibiotics has shown good performance in the early battle with *H. pylori* infection. With the widespread application of antibiotics in clinical practice, *H. pylori* resistance to antibiotics has increased to different degrees worldwide. To solve this problem, the treatment plan for *H. pylori* infection has changed from initial triple therapy to quadruple therapy, and the treatment period has been gradually extended, which seriously affects patient compliance as well as the quality of life [5, 7]. However, the success rate of *H. pylori* eradication is still declining, and *H. pylori* eradication is now becoming a difficult challenge for clinical physicians [8]. Moreover, the recurrence of *H. pylori* infection, including the recrudescence and reinfection of *H. pylori*, is also of concern; the recurrence rate is estimated to be 10.9% of patients after eradication treatment in developing countries, and quadruple therapy is required for most recurrent cases to reeradicate *H. pylori* infection [9–11].

Previous studies have shown that *H. pylori* infection causes gastrointestinal microbiota disorder, and this change is reversible after *H. pylori* eradication [12–14]. In addition, antibiotic-based treatments for *H. pylori* eradication have been shown to cause gut microbiota dysbiosis and lead to the increase of *erm (B)* gene (a gene encoding erythromycin-resistant methylase), which would compromise the efficacy of eradiation therapy regimens including a macrolide [12, 15, 16]. Therefore, it may be possible to reverse the colonization of *H. pylori* by restoring the gastrointestinal microbiota. It has been proven that supplementation of probiotics such as *Lactobacillus acidophilus* and *Saccharomyces boulardii* in traditional triple therapy can effectively improve the eradication rate of *H. pylori* infection and reduce the incidence of adverse events. However, there are still no probiotics that can be used alone to eradicate *H. pylori* infection [17, 18].

Fecal microbiota transplantation (FMT), in which the fecal microbiota of a healthy individual is transplanted into the patient's intestines, has been shown to effectively restore the gastrointestinal microbiota and treat gastrointestinal diseases. It has been demonstrated that FMT is efficacious for the treatment of a variety of gut microbiota-related diseases, including digestive system and nondigestive system diseases [19–22]. FMT on the basis of washed microbiota preparation, known as washed microbiota transplantation (WMT), has been proven to decrease adverse events caused by traditional fecal suspension preparation and greatly improve the efficacy [23, 24]. We speculate that WMT can also be used, alone or in combination with currently recommended regimens, to eradicate *H. pylori* infection, by restoring the gut microbiota. However, this hypothesis has not been tested.

Therefore, the aim of this pilot study was to explore the feasibility and efficacy of WMT on *H. pylori* eradication.

## 2. Materials and Methods

### 2.1. Patients

Consecutive patients, who had been treated with WMT for various indications at the First Affiliated Hospital of Guangdong Pharmaceutical University (Guangdong, China) and were positive for *H. pylori* infection within 1 year before WMT treatment but had not been treated with any eradication therapy for *H. pylori* infection during the period, were identified by reviewing the hospitalization data. Then, a telephone call was conducted, and those patients who were not receiving eradication therapy for *H. pylori* infection after WMT were invited to take a follow-up ^13^C-urea breath test (UBT), which was performed at least 4 weeks after the completion of WMT and withdrawal of proton pump inhibitors and antibiotics. Only patients who took the follow-up examination were included in the final analysis.

Gastric function indicators including pepsinogen I (PGI), pepsinogen II (PGII), pepsinogen ratio (PGR), gastrin 17 (G-17), and intestinal barrier function indicators including diamine oxidase (DAO), D-lactate, and lipopolysaccharide (LPS) detected the week before and after WMT were analyzed.

The protocol of this study was approved by the Ethics Committee of the First Affiliated Hospital of Guangdong Pharmaceutical University, and all patients who took the follow-up ^13^C-UBT provided written consent according to the Measures for Ethical Review of Biomedical Research Involving Human Beings (http://www.gov.cn/gongbao/content/2017/content_5227817.htm).

### 2.2. WMT

#### 2.2.1. Stool Donors

The methods for donor screening and stool suspension preparation were consistent with the Nanjing Consensus on Methodology of Washed Microbiota Transplantation [24]. The donors' ages ranged between 18 and 25 years old, and their body mass indexes were between 18.5 and 23.9. All donors needed to pass a structured questionnaire firstly, and those who met the requirements were invited to participate in further interview and psychological and physical examinations. Donors with infectious diseases, digestive diseases, metabolic diseases, chronic fatigue syndrome, autoimmune diseases, allergic disease, and neuropsychiatric diseases were excluded. All qualified donors were required to receive a training about healthy diet prior to stool donation.

#### 2.2.2. Stool Suspension

The stool samples provided by the donor were collected, weighed, added with sterile saline according to the ratio of feces to saline (1 : 5), and then mixed evenly. The mixture was filtered through the intelligent microbial separation system (GenFMTer; FMT Medical, Nanjing, China), and five stages of filtration were carried out. The obtained suspension was then immediately centrifuged at a speed of 2500 rpm for 3 minutes and repeated three times. The final sediment was suspended again with sterile saline in accordance with the ratio of sediment to saline (1 : 1).

#### 2.2.3. WMT Preoperative Preparation

Metoclopramide was injected intramuscularly 30 minutes before WMT, and a proton pump inhibitor (Omeprazole or Lansoprazole) was injected intravenously one hour before WMT.

#### 2.2.4. WMT Procedure

Before WMT, an endoscopic administration tube (nasojejunal or transendoscopic enteral tube) was placed in the stomach (or upper) or jejunum (or middle) along the upper gastrointestinal tract or in the right hemicolon (or lower) along the lower gastrointestinal tract and fixed with titanium clips with the assistance of a gastroscope or enteroscope, and then, the endoscopic administration tube was flushed with normal saline to confirm the patency [25]. The gastrointestinal tract delivery route was dependent on the patient's wish or tolerance. The stool suspension was infused according to the standard of 200 mL per person. Finally, the patient was asked to stay in the lying position for 30 min, with restriction of strenuous exercise. During the course of treatment, the frequency of WMT was once a day for three consecutive days, and the actual course patients received was adjusted according to the patient's condition.

### 2.3. Detection of H. pylori Infection

^13^C-UBT was used for the *H. pylori* detection before and during WMT for some patients and at least 4 weeks after the completion of WMT for all included patients. During the test, the patient's first breath was collected after fasting for 2 h, followed by oral administration of ^13^C-urea (Beijing Boran Pharmaceutical Co., Ltd., Beijing, China), and the second breath was collected 30 min later. The values of CO_2_ at baseline and 30 min later were measured by an isotope ratio mass spectrometer (Beijing Richen-Force Science & Technology Co., Ltd., Beijing, China). Positivity was defined when the ^12^C/^13^C ratio (*δ* value) was greater than 4 in the breath sample before and after administration of the ^13^C-urea [26].

A commercial enzyme-linked immunosorbent assay kit (Beijing Wantai DRD Co., Ltd., Beijing, China) was used to detect the *H. pylori* antibody, and the *H. pylori* infection status was diagnosed according to the manufacturer's instructions [19]. For patients who underwent upper endoscopy, gastric specimens obtained by endoscopy were embedded in paraffin and sectioned, followed by hematoxylin and eosin staining for histological examination and the rapid urease test [27]. Patients with dark blue arcs or S-shaped bacteria in the section under the light microscope and/or with color change in the reagent (Fujian Sanqiang Biochemical Co., Ltd., Fujian, China) were considered *H. pylori* positive.

In this study, *H. pylori* status before or during WMT was defined as positive when any one of the four tests (*i.e.*^13^C-UBT, serology test for *H. pylori*-IgG antibody, rapid urease test, pathological examination) was positive. To further strengthen the quality of the study, further analysis for patients who tested positive for *H. pylori* infection by at least two tests or at least twice by a single test within 1 year before WMT was performed. *H. pylori* eradication was defined as a negative *H. pylori* status in the follow-up ^13^C-UBT at least 4 weeks after the completion of WMT.

### 2.4. Data Analysis

Categorical data are expressed as the frequency and percentage and numerical data as the mean ± standard deviation. The enumeration data were tested by the chi-square test and Fisher's exact test; the odds ratio (OR) and 95% confidence interval were calculated. If a normal distribution was obeyed, Student's *t*-test was used for the comparison between the groups; otherwise, the Kruskal-Wallis *H* test was used for replacement. In addition, the variables with *P* < 0.20 were analyzed in multivariate logistic regression analysis. SPSS software version 19.0 (IBM, Armonk, NY, USA) was used to analyze the data. The difference was defined as statistically significant when *P* < 0.05.

## 3. Results

### 3.1. Effect of WMT on H. pylori Infection

**Figure 1:**
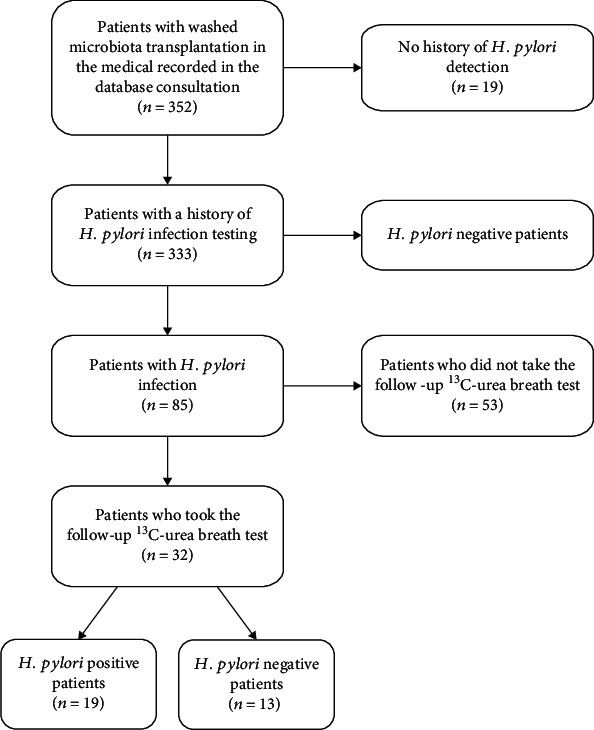
Flowchart for the inclusion of patients.

A total of 352 hospitalized patients who received WMT were identified, of whom 19 did not have a history of *H. pylori* detection and 248 were *H. pylori*-negative. Thus, 85 H*. pylori*-positive patients before and during WMT were further reviewed. Of the 85 *H. pylori*-infected patients, only 32 had a ^13^C-UBT after WMT and did not receive *H. pylori* eradication therapy (Figure 1). Finally, 32 patients, including 19 (59.4%) males and 13 (40.6%) females, were included in the analysis. Among these patients, 13, 5, 5, and 2 patients were diagnosed with *H. pylori* infection by a single *H. pylori*-serology test alone, ^13^C-UBT alone, rapid urease test alone, and pathological examination alone, respectively, and the remaining seven patients were diagnosed with *H. pylori* infection by at least two tests (*n* = 6) or at least twice by a single test (*n* = 1). The average age of these patients was 57.22 ± 18.29, ranging from 9 to 86 years. Irritable bowel syndrome (IBS) was the most common indication for WMT, accounting for 59.38% (*n* = 19), followed by nonalcoholic fatty liver disease (NAFLD), hepatic encephalopathy (HE), gastroesophageal reflux disease (GERD), gouty arthritis (GA), alcoholic hepatitis (AH, all 6.25%, *n* = 2), hepatic cirrhosis (HC), functional dyspepsia (FD), and attention deficit hyperactivity disorder (ADHD, all 3.13%; *n* = 1). Of the 32 patients, 13 (40.6%) became negative, while the other 19 (59.4%) remained positive for *H. pylori* infection in the ^13^C-UBT after WMT. The 13 patients became *H. pylori* negative, one patient used moxifloxacin for urinary tract infection for 1 week, five had never used any antibiotics, and seven were unsure or could not remember whether they used antibiotics during the one year prior to WMT. In addition, of the seven patients who were positive for *H. pylori* infection by at least two tests or at least twice by a single test within 1 year before WMT, two (28.57%) became negative at the follow-up visit.

### 3.2. Associations of WMT with Therapeutic Effects

There was no significant difference in the eradication rate between male and female patients (31.58% *vs.* 53.85%, OR = 0.396, 95% CI: 0.92–1.699, *χ*^2^ = 1.587, *P* = 0.208), and among those with different indications (26.3%, 100%, 50%, 100%, 0%, 100%, 100%, 0%, and 0% for IBS, NAFLD, HE, GERD, GA, AH, HC, FD, and ADHD, respectively; *P* = 0.120) (Table 1). The rate was not significantly different among patients who received WMT via upper, middle, and/or lower gastrointestinal tract delivery route; however, the rate with middle gastrointestinal tract delivery route only appeared to be higher than that with lower gastrointestinal tract delivery route only (58.33% *vs* 16.67%, OR = 7, 95% CI: 0.613–79.871; *P* = 0.152). There was no significant difference in the age (60.38 ± 14.25*vs.*55.00 ± 20.67 years; *P* = 0.422), course times of WMT (2.46 ± 1.13*vs*.2.74 ± 1.66 times; *P* = 0.607), frequencies of WMT (7.15 ± 3.44*vs.*8.32 ± 4.97 times; *P* = 0.471), and duration from the completion WMT to last ^13^C-UBT (428.23 ± 262.17*vs.*600.89 ± 424.31 days; *P* = 0.202) between patients with and without *H. pylori* eradication (Table 1). Data were available on intestinal barrier function for all patients and on gastric function for 31 patients (one patient who remained positive for *H. pylori* infection did not undergo the test). There were no significant differences in the values of DAO (6.03 ± 6.00 U/L *vs.*4.92 ± 2.97 U/L), D-lactate (13.06 ± 6.913 U/L *vs.*13.91 ± 8.43 U/L), LPS (7.06 ± 8.65 U/L *vs.*9.82 ± 7.18 U/L), PG I (126.33 ± 56.73 *μ*g/L *vs.*138.44 ± 88.39 *μ*g/L), PG II (12.10 ± 7.27 *μ*g/L *vs.*19.66 ± 15.51 *μ*g/L), and G-17 (5.65 ± 11.36 pmol/L *vs.*8.02 ± 11.45 pmol/L) between patients with and without *H. pylori* eradication.

The PGR was significantly higher in patients with *H. pylori* eradication than those in whom *H. pylori* infection was persistent (13.00 ± 6.97*vs.*8.31 ± 3.73; *P* = 0.022). However, the subsequent multivariate logistic regression analysis did not show any association between pre-WMT PGR and the efficacy of WMT (OR = 1.152, 95%: 0.959-1.384, *P* = 0.130).

## 4. Discussion

In this study, we found that 40.6% of patients who received WMT had *H. pylori* infection eradicated after the treatment, which was significantly associated with an increased pre-WMT PGR but not with patient age, gender, indications, delivery route, course and frequency of WMT, and the intestinal function.

In the present study, we found that WMT, a microbial therapy, had a certain efficacy on *H. pylori* infection. If confirmed, it is undoubtedly a breakthrough for the traditional eradication therapy that relies significantly on antibiotics, as WMT can be used as a direct or indirect means for *H. pylori* infection. Although *H. pylori* infection can be eradicated by triple or quadruple antibiotic-based therapy in over 80% of patients, the problem of *H. pylori* resistance has gradually emerged with the extensive use of antibiotics [5, 7]. The multidrug resistance rate of *H. pylori* varies from 10% to 40%, and even sextuple resistance has been detected in some countries [28]. The increased antibiotic resistance in *H. pylori* will further reduce patients' quality of life and increase the cost-effectiveness of antibiotic-based therapy [8]. In addition, prolonged eradication therapy for *H. pylori* infection also leads to dysbiosis of the intestinal microbiota and increases the expression of resistance genes, which may further induce various diseases [12, 15, 16, 29]. However, with the emergence of WMT and fecal suspension capsules, the safety and convenience of WMT have been significantly improved [23, 30]. It is notable that WMT may be used for refractory *H. pylori* infection (defined as those who have failed the first eradication treatment or with recurrence of *H. pylori* infection). Chen et al. found that *Lactobacillus rhamnosus* and *Lactobacillus acidophilus* had effective antimicrobial activity against multidrug-resistant *H. pylori* by inhibiting *H. pylori*-induced inflammation and promoting the growth of probiotics [31]. WMT, which is also a microbial therapy, may affect the colonization of *H. pylori* by increasing the abundance of the above probiotics and reducing the inflammation caused by refractory *H. pylori* infection. Previous studies have shown that WMT has better cost-effectiveness in treating some recurrent diseases such as *Clostridium Difficile* infection and inflammatory bowel disease, compared with conventional therapies [32, 33]. Therefore, WMT may also be a good alternative to antibiotics for the eradication of refractory *H. pylori* infection. Due to the limitation of clinical data, we were unable to explore the role of WMT for refractory *H. pylori* infection in this pilot study. However, it is worthy of further in-depth investigation.

We observed that the success of WMT in eradicating *H. pylori* infection appeared to be associated with an increased PGR detected within 1 week prior to WMT. It has been demonstrated that a low PGR is a biomarker of precancerous lesions, such as atrophic gastritis, and thus indicates a high risk of developing gastric cancer [34–36]. Our observation suggests that WMT may have a therapeutic effect on *H. pylori* infection in patients with low risk of gastric cancer and those with high risk of gastric cancer may not benefit from WMT. Therefore, PGR may be used as one of the evaluation indicators for microbial intervention in the treatment of *H. pylori* infection. Further investigation is required to elucidate the mechanism for the favorite therapeutic effect of WMT in patients with a high PGR.

It is noticeable that female patients appeared to be more likely to have a higher eradication rate of *H. pylori* infection than male patients after WMT, which may be related to the differences in hormone levels. Hosoda et al. [37] found that some steroid hormones, including estradiol, androstenedione and progesterone, were effective in the inhibition of *H. pylori* infection, suggesting that high levels of estradiol, androstenedione, and progesterone in female patients may enhance the therapeutic effect of WMT in eradicating *H. pylori* infection. However, further investigation is required to determine whether the efficacy of WMT in eradicating *H. pylori* infection is better in females than in males and the underlying mechanism.

Four patients underwent WMT *via* the upper gastrointestinal tract (gastric) delivery route in the present study, but they had also received WMT *via* the middle gastrointestinal tract (small intestine) delivery route at the same time. Thus, we were unable to compare the difference in the efficacy between the three delivery routes. However, patients who received WMT *via* middle gastrointestinal tract delivery route alone appeared to have a higher eradication rate than those with a lower gastrointestinal tract delivery route although the difference was not statistically different, most likely due to the small number of cases in this preliminary study. The ability of the translated fecal microbiota to spread and colonize into the stomach may contribute to the difference in the eradication rate. *H. pylori* specifically colonizes in the stomach, where the low pH is hostile to the growth of other microbiota [3, 38]. Among the three delivery routes, the distance between the lower route (*i.e.*, the right hemicolon) and the stomach is the longest, and the pH of the upper route (*i.e.*, the stomach *per se*) is the lowest; thus, the fecal microbiota translated through these two routes may be difficult either to reach the stomach or to hardly survive in the low-pH environment of the stomach. Therefore, the middle route (*i.e.*, the jejunum) may be the most favorable location for the translated fecal microbiota to adapt, spread, and colonize the stomach, whereby exhibiting anti-*H. pylori* effects. However, many patients in the present study received several courses of WMT through more than one sites since the original purpose of WMT was for indications other than *H. pylori* infection; we could not determine the optimal delivery route. Therefore, well-designed studies with a large number of patients are needed to observe the optimal delivery route for the treatment of *H. pylori* infection with WMT.

It should be mentioned that although all patients enrolled in this study had never received *H. pylori* eradication therapy, some patients had received antibiotics for various conditions within one year prior to WMT. However, they did not take two or more antibiotics simultaneously and the duration of antibiotic use did not last for more than a week. It is generally accepted that successful eradication of *H. pylori* infection can only be achieved by a combined administration of at least two antibiotics in combination with a proton pump inhibitor with or without a bismuth salt, for 7–14 days [39]. It is rare, if any, that a single antibiotic can successfully eradicate *H. pylori* infection. To further confirm the efficacy of WMT for *H. pylori* infection, we analyzed the therapeutic effect of WMT in the patients who were positive for *H. pylori* infection by at least two tests or at least twice by a single test before WMT and observed that the eradication rate remained 28.57%. A serology test for IgG anti-*H. pylori* antibody was used as one of the diagnostic methods in the present study due to its high accuracy in the diagnosis of *H. pylori* infection [40]. It has been demonstrated that the existence of IgG anti-*H. pylori* antibody in patients represents current active *H. pylori* infection if the patients have never received any eradication therapy, which is the case in the present study, as spontaneous elimination of *H. pylori* infection in adults is extremely rare [41]. There might be a possibility that false-negative post-WMT UBT results were obtained in some patients. However, ^13^C-UBT has been used in clinical practice as a standard method to determine *H. pylori* status after *H. pylori* eradication therapy, due to its high sensitivity and specificity [42–44]. Thus, it is unlikely that the false-negative results ^13^C-UBT occurred in nearly 30% of cases after WMT. Therefore, we believe that our observation was not opportunistic. It should be acknowledged that the sample size of the present study was relatively small, due to the stringent inclusion criteria of this study, which may affect the accuracy of the study results to a certain extent. However, the present study, for the first time, demonstrates the therapeutic efficacy of WMT for *H. pylori* infection and thus provides a novel direction for searching regimens with promising efficacy in the eradication of *H. pylori* infection. In the future, more attention should be paid to optimization of WMT, confirmation of the efficacy, as well as safety, of WMT, especially in combination with currently recommended regimens for *H. pylori* infection in randomized controlled trials, determination of the influencing factors, and elucidation of the underlying potential mechanisms.

## 5. Conclusion

*H. pylori* infection was eradicated in a proportion of patients who received WMT. An increased pre-WMT PGR appeared to be associated with the therapeutic effect of WMT. Further clinical studies are required to confirm the efficacy, as well as safety, of WMT, especially in combination with currently recommended regimens in randomized controlled trials.

## Figures and Tables

**Table 1:** Associations of age, gender, indications, and procedures of WMT with *H. pylori* eradication by WMT.

Variable	*H. pylori* eradicated	*H. pylori* persistent	*P* value
Age	60.38 ± 14.25	55.00 ± 20.67	0.422
Gender			
Male (*n* = 19)	6 (31.58)	13 (68.42)	0.208
Female (*n* = 13)	7 (53.85)	6 (46.15)	
Indications			
IBS (*n* = 19)	5 (26.32)	14 (73.68)	0.120
NAFLD (*n* = 2)	2 (100.00)	0 (0.00)	
HE (*n* = 2)	1 (50.00)	1 (50.00)	
GERD (*n* = 2)	2 (100.00)	0 (0.00)	
GA (*n* = 2)	0 (0.00)	2 (100.00)	
AH (*n* = 2)	2 (100.00)	0 (0.00)	
HC (*n* = 1)	1 (100.00)	0 (0.00)	
FD (*n* = 1)	0 (0.00)	1 (100.00)	
ADHD (*n* = 1)	0 (0.00)	1 (100.00)	
Delivery route^∗^			
Middle gastrointestinal tract only (*n* = 12)	7 (58.33)	5 (41.67)	0.152
Lower gastrointestinal tract only (*n* = 6)	1 (16.67)	5 (83.33)	
Upper gastrointestinal tract and middle gastrointestinal tract (*n* = 4)	2 (50.00)	2 (50.00)	
Middle and lower gastrointestinal tract (*n* = 8)	3 (37.50)	5 (62.50)	
Upper, middle and lower gastrointestinal tract (*n* = 2)	0 (0.00)	2 (100.00)	
WMT procedures			
Course times	2.46 ± 1.13	2.74 ± 1.66	0.607
Frequency	7.15 ± 3.44	8.32 ± 4.97	0.471
Duration (day)	428.23 ± 262.17	600.89 ± 424.31	0.202
Intestinal barrier function (*n* = 32)			
Diamine oxidase (U/L)	6.03 ± 6.00	4.92 ± 2.97	0.545
D-lactate (U/L)	13.06 ± 6.913	13.91 ± 8.43	0.768
Lipopolysaccharide (U/L)	7.06 ± 8.65	9.82 ± 7.18	0.332
Gastric function (*n* = 31)			
PG I (*μ*g/L)	126.33 ± 56.73	138.44 ± 88.39	0.669
PG II (*μ*g/L)	12.10 ± 7.27	19.66 ± 15.51	0.114
PG ratio (PG I/PG II)	13.00 ± 6.97	8.31 ± 3.733	0.022
Gastrin-17 (*μ*g/L)	5.65 ± 11.36	8.02 ± 11.45	0.573

Data are expressed as the mean with standard deviation or number (%), where appropriate. ^∗^During WMT, an endoscopic administration tube was placed in the stomach (or upper) or jejunum (or middle) through the upper gastrointestinal tract or right hemicolon (or lower) through the lower gastrointestinal tract. *P* = 0.152, compared between patients receiving WMT *via* middle gastrointestinal tract delivery route alone and those with lower gastrointestinal tract delivery route alone. IBS: irritable bowel syndrome; NAFLD: nonalcoholic fatty liver disease; HE: hepatic encephalopathy; GERD: gastroesophageal reflux disease; GA: gouty arthritis; AH: alcoholic hepatitis; HC: hepatic cirrhosis; FD: functional dyspepsia; ADHD: attention deficit hyperactivity disorder; PG: pepsinogen.

## Data Availability

The [DATA TYPE] data used to support the findings of this study are available from the corresponding author upon request.
